# Presence of diastolic dysfunction after biphasic synchronized transthoracic shocks in a porcine model evaluated with CMR

**DOI:** 10.1186/1532-429X-16-S1-P81

**Published:** 2014-01-16

**Authors:** Gobinath Nadeshalingam, Dominik P Guensch, Janelle Yu, Kady Fischer, Matthias G Friedrich

**Affiliations:** 1 Philippa & Marivn Carsley CMR Centre, Montreal Heart Institute, Montreal, Quebec, Canada; 2 Anesthesiology and Pain Medicine, University Hospital Bern, Bern, Switzerland

## Background

Defibrillation and cardioversion are often used as life-saving measures in cases of cardiac arrest and arrhythmias. Previous studies in humans with cardioversion of arrhythmias, found a decrease in ejection fraction but no significant changes in preload, afterload and heart rate. However, it is not clear whether the underlying cardiac condition may have contributed to these findings. This study aimed to observe the effects of biphasic synchronized shocks on left-ventricular function parameters over a 5-hour period in a porcine model.

## Methods

Ten pigs received five consecutive biphasic synchronized shocks of 200J. Six pigs served as healthy controls and underwent the identical anesthesia and imaging protocol. Images were acquired with a clinical 3T MRI scanner (Siemens Magnetom Skyra). Routine functional cine imaging was completed for all pigs at baseline by acquiring a short-axis stack (7-10 slices) of the left ventricle. This imaging protocol was repeated hourly for 5 hours. All MR images were analyzed for cardiac function parameters (cardiac output, stroke volume, ejection fraction, end-diastolic and end-systolic volume) and in addition to assess left ventricular motion abnormalities. T2 maps were acquired at each time point to evaluate the presence of myocardial edema following the shock series.

## Results

**Figure 1:**
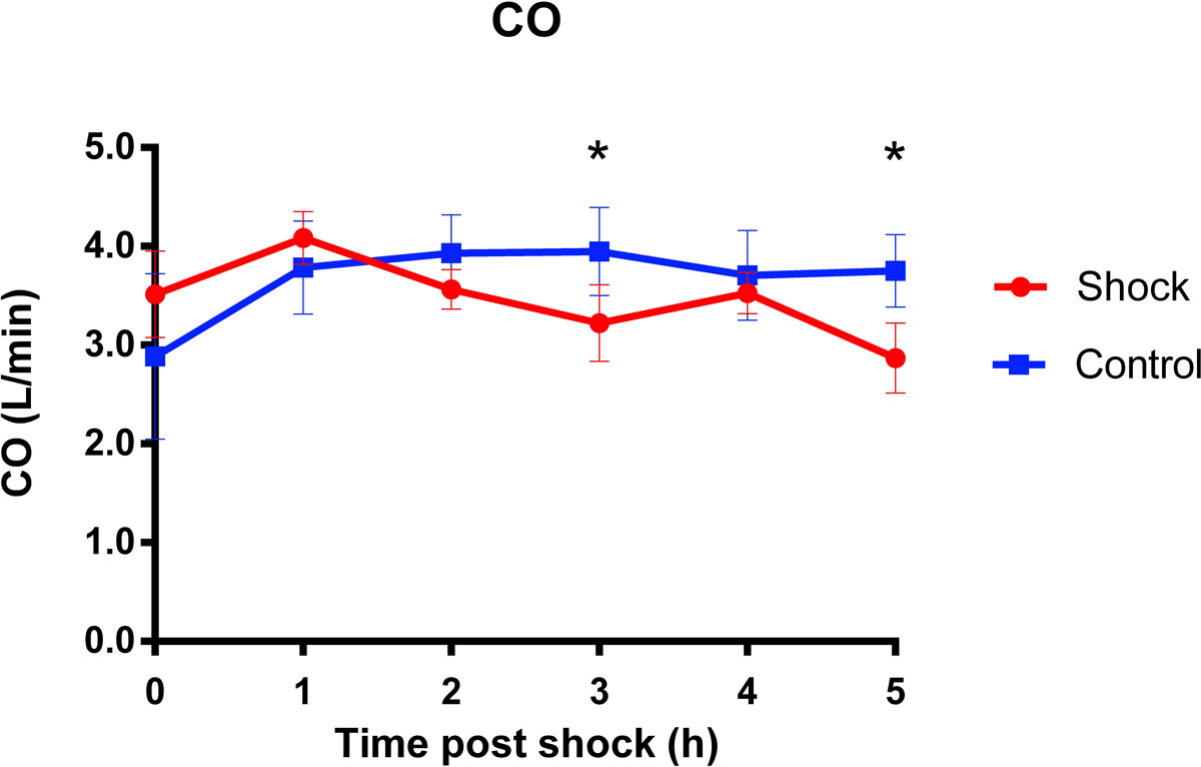
**Mean ± SEM change in cardiac output (CO) in the shock and control group**. There was a significant decrease in CO in the shock group (*p < 0.05 vs. baseline).

**Figure 2:**
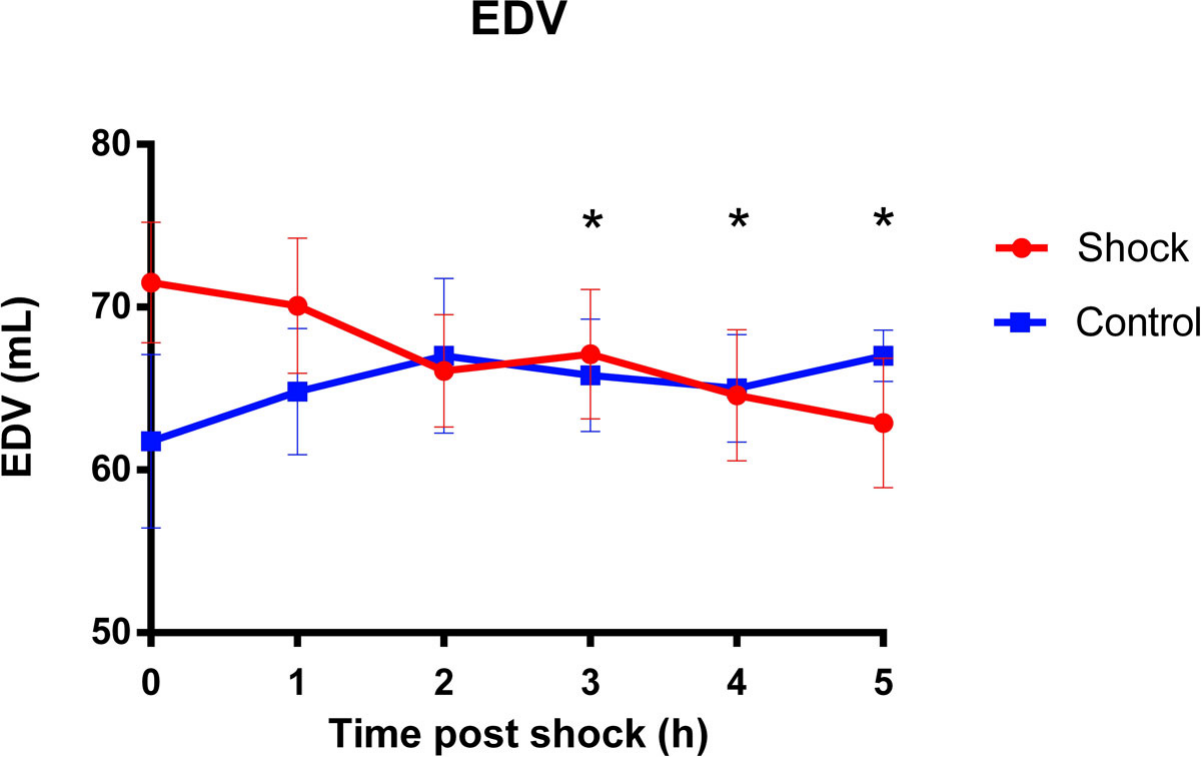
**Mean ± SEM change in left ventricular end-diastolic volume (EDV) in the shock and control group**. There was a significant decrease in EDV in the shock group (*p < 0.05 vs. baseline).

Four out of ten pigs required pharmacological vasopressor support with Phenylephrine or Noradrenaline within the first hour after the shocks to keep blood pressure stable (MAP >50 mmHg), while no vasopressors were required in the control group. A significant decrease in cardiac output (CO) from baseline (4.00 ± 0.24 L/min) was observed that was significant at 3 and 5 hours post-shock, (3.57 ± 0.19 and 3.15 ± 0.19 L/min respectively; p < 0.05, Figure 1). End-diastolic volume (EDV) decreased significantly from baseline (72.33 ± 4.05 mL) at 3-5 hours post shock to a minimum of 62.89 ± 3.97 mL in the shock group (p < 0.05, Figure 2). The assessment of left ventricular wall motion indicated a significant reduction in wall thickening during contraction in the septum at 3 hours post shock. A small global increase in T2 was observed in the left ventricle (1.41 ± 2.83%), which was significantly different from a decreased T2 in the control group (-6.3 ± 2.15%; p < 0.05) at 3 hours post-shock, consistent with a higher myocardial water content. No significant changes in heart rate were observed between the control and defibrillation groups. There was no difference in fluid administration (2.3 vs. 2.1L) and anesthetic doses (21.89 vs. 26.21 mg/kg/h Propofol) between the defibrillation and control group, respectively.

## Conclusions

Biphasic synchronized shocks of cumulative 1000J in a porcine model lead to a significant decrease in EDV and CO. Shock-induced myocardial edema may be an explanation of our findings indicating diastolic dysfunction with preserved ejection fraction.

## Funding

Funding is provided by the Montreal Heart Institute Foundation and the Canadian Foundation for Innovation.

